# Organogels Fabricated from Self-Assembled Nanotubes Containing Core Substituted Perylene Diimide Derivative

**DOI:** 10.1021/acsomega.2c02210

**Published:** 2022-06-14

**Authors:** Prajna Moharana, G. Santosh

**Affiliations:** Division of Chemistry, School of Advanced Sciences, Vellore Institute of Technology, Chennai 600127, India

## Abstract

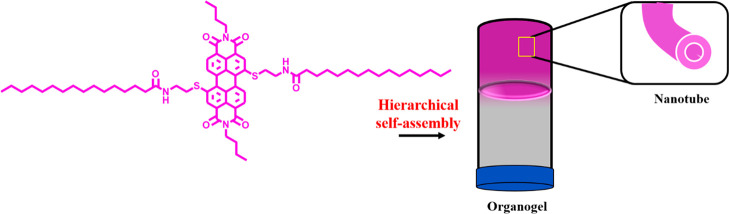

Perylene-based organogels are well-known for their applications as sensors and optoelectronic materials. Among them, core-substituted perylene diimide-based organogels are rarely explored. Herein, the hierarchical self-assembly mechanism of a newly synthesized, amide-linked core-substituted perylene diimide derivative, which formed organogels in organic solvents like toluene and methyl cyclohexane (MCH), is discussed. These organogels are composed of one-dimensional molecular aggregates like nanofibers and nanotubes. Organogels composed of nanofibers are very frequent. On the contrary, for the first time, we have encountered a perylene diimide-based organogel consisting of self-assembled nanotubes. The molecular interactions, molecular packing, and rheological properties of this organogel are also discussed.

## Introduction

In the past few years, low-molecular-weight supramolecular organogels^1−7^ have been proven to be useful materials in the field of biotechnology and the materials world. Because of their extraordinary supramolecular architectures, they have the potential to act as sensors and light-harvesting materials.^8^ They are also sensible materials for the fabrication of optical devices.^9^ These supramolecular architectures are constructed by hierarchical self-assembly, where non-covalent interactions like hydrogen bonding and π–π interactions play a vital role to drive the molecules toward aggregation.^10,11^

Perylene tetracarboxylic diimides (PDI) are found to be excellent building blocks for constructing supramolecular architectures because of their extended π-conjugation.^10,12−16^ PDI-based organogels are promising materials for fabricating electronic and optical devices.^17−23^

Core-substituted PDI-based organogels are rarely reported in the literature. Wurthner and his co-workers have reported a fluorescent organogel of PDI containing a phenoxy group at its core positions.^24^ Yagai and co-workers have prepared stimuli-responsive soft materials of PDI-functionalized flexible bisurea in several chlorinated solvents.^25^ It is well-known that these organogels consist of fiber structures, which are necessary for the gelation. However, the PDI-based organogels composed of nanotubes have not been reported so far.

**Scheme 1:**
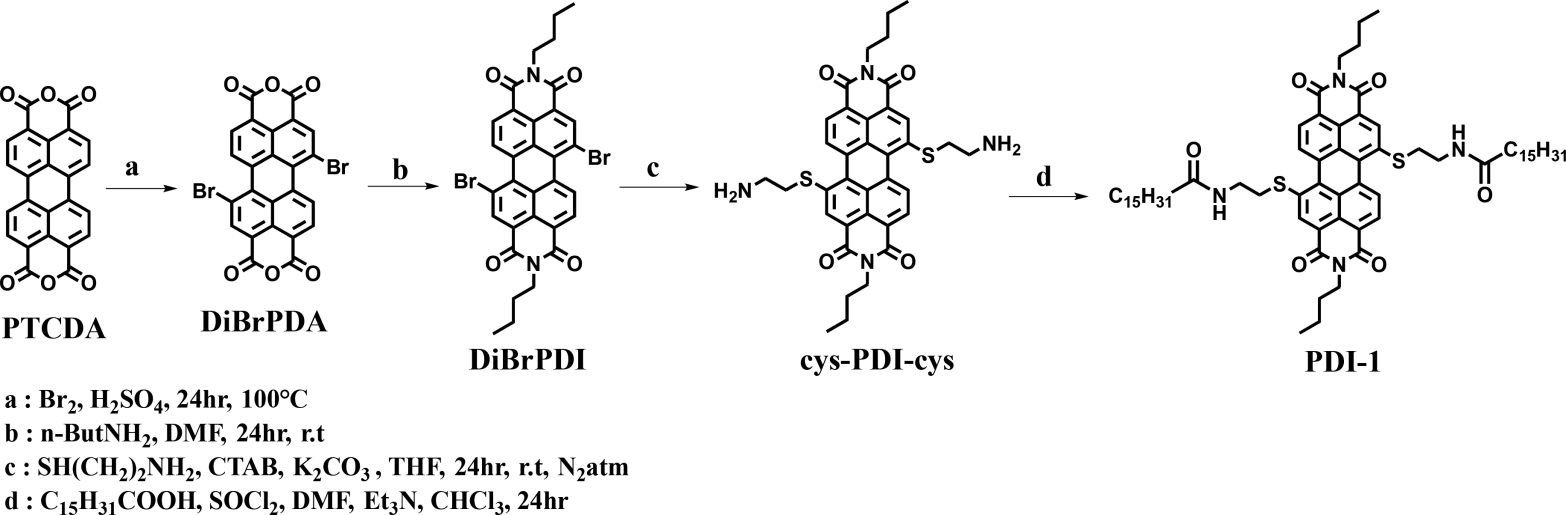
Synthesis of **PDI-1** Br_2_, H_2_SO_4_, 24 h, 100 °C *n*-ButNH_2_, DMF, 24 h, r.t SH(CH_2_)_2_NH_2_, CTAB, K_2_CO_3_, THF, 24 h, r.t, N_2_atm C_15_H_31_COOH, SOCl_2_, DMF, Et_3_N, CHCl_3_, 24 h

Here, we disclose organogels composed of both nanotubes and nanofibers from a new core-substituted **PDI-1** (Scheme 1). A new core-substituted PDI was synthesized, and its self-assembly studied by spectroscopic methods. The gelation ability of **PDI-1** was tested and was found to form gels in toluene and methyl cyclohexane (MCH). These gels were composed of nanotubes^16,26,27^ and nanofibers, respectively, and were analyzed by electron microscopy. These structures were studied using the powder X-ray diffraction (PXRD) technique, and infrared (IR) spectroscopy. It was found that the involvement of hydrogen-bonding directed π–π interaction of perylene cores in intermolecular hierarchical self-assembly leads to gelation in different organic solvents.

## Results and Discussion

### Molecular Design and Synthesis

**PDI-1** containing a long alkyl chain through an amide linkage at the core positions was synthesized in good yields according to the methods discussed in Scheme 1. The commercially available perylene tetracarboxylic dianhydride (PTCDA) was used as the starting material for the synthesis of 1,7-dibromo perylene dianhydride (DiBrPDA), followed by conversion to diimides using butylamine (DiBrPDI).^28−30^ Nucleophilic substitution of bromine in 1,7-dibromo perylene diimide (DiBrPDI) by the sulfur of cysteamine at the core positions led to the formation of the new intermediate (cys-PDI-cys). This intermediate was then condensed with palmitic acid affording the **PDI-1** in 69% yield. The identity of cys-PDI-cys and **PDI-1** was confirmed by ^1^H NMR, high-resolution mass spectrometry (HR-MS), and MALDI-TOF mass spectrometry.

### Self-Assembly Studies

**Figure 1:**
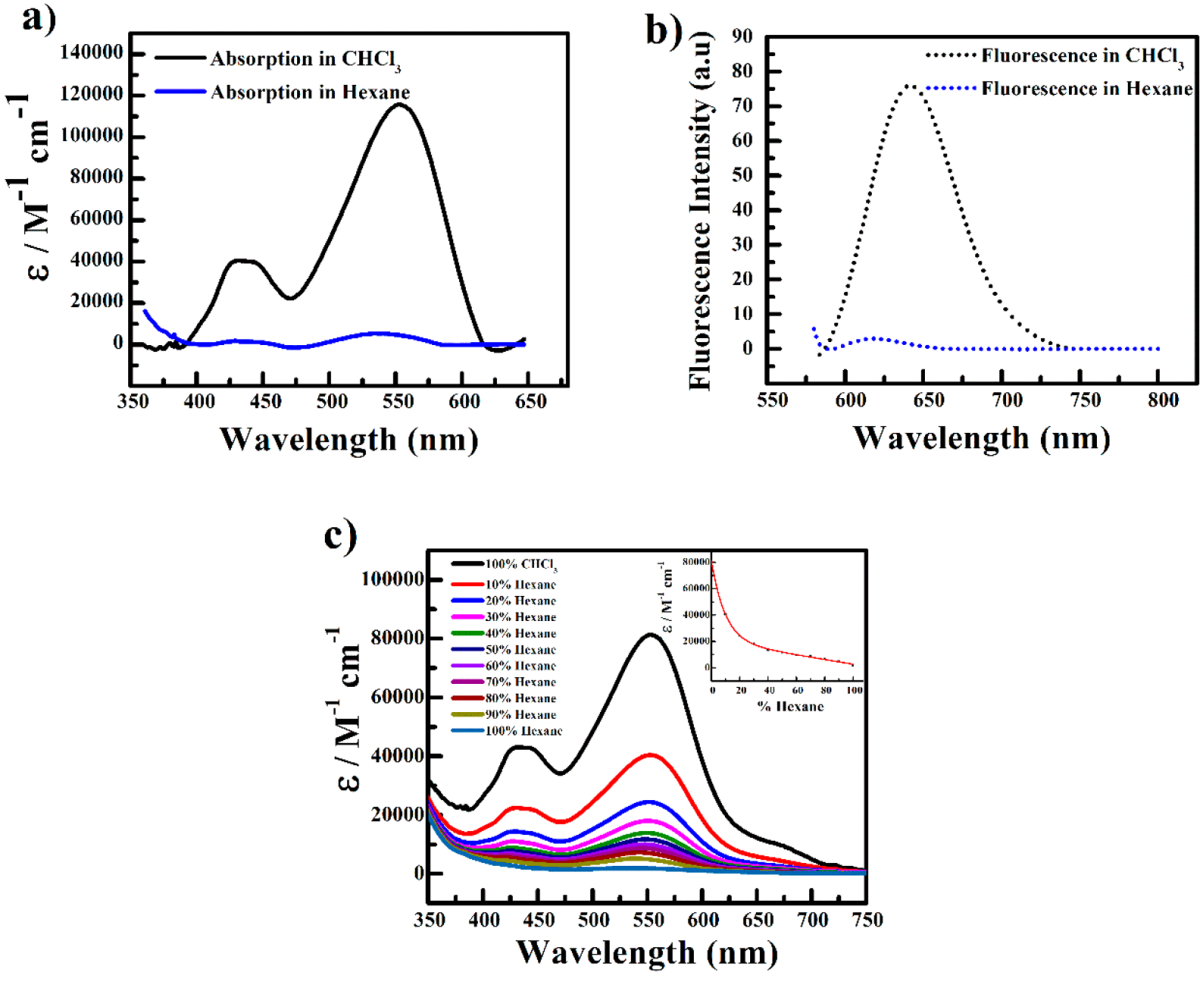
(a) Absorption and (b) fluorescence spectra of **PDI-1** in CHCl_3_ and hexane (5 μM). (c) Absorption spectra of **PDI-1** (5 μM) in increasing volume ratio of CHCl_3_/hexane (inset: absorption intensity at λ _max_ = 553 vs volume of hexane in chloroform solution).

The UV–vis spectroscopic studies of **PDI-1** were performed in various polar and nonpolar organic solvents (Figure S3). **PDI-1** was found to be readily soluble in CHCl_3_ and showed a pronounced absorption peak at λ_max_ = 553 nm, with a shoulder around 433 nm corresponding to 0–0 and 0–1 vibronic transitions, respectively (Figure 1a).^9,31,32^ The corresponding fluorescence spectrum showed a peak at λ_em_ = 640 nm (Figure 1b).^31^ These significant peak positions are the indication of the disaggregated form of **PDI-1** in CHCl_3_ solution.^32^**PDI-1** is partially soluble in nonpolar solvents like toluene, MCH, and hexane, indicating that it may tend to aggregate in these solvents. As summarized in Table 1, **PDI-1** formed *H*-*type* aggregates in hexane, toluene, and MCH as indicated by the blue-shift of λ_max_ and λ_em_ by a few nanometers from their disaggregated form in CHCl_3_. The formation of these *H*-*type* aggregates was also evidenced from the drop in the fluorescence intensity compared with that of the CHCl_3_ solution (Figure S4).

**Table 1:** Absorption and Fluorescence Data for **PDI-1** in Various Solvents

s_no.	solvents	concentration	λ_max_ (nm)	shoulder peak	λ_em_ (nm)	remark
1	chloroform (CHCl_3_)	5 μM	553	433	640	disaggregated
2	hexane	5 μM	533	432	619	aggregated
3	methyl cyclohexane (MCH)	5 μM	536	433	613	aggregated
4	toluene	5 μM	550	436	631	aggregated

In hexane, the absorption spectrum of **PDI-1** showed major changes compared with their disaggregated form in CHCl_3_. Both λ_max_ and λ_em_ showed a strong blue-shift of 20 nm with a maximum drop in absorption intensity and quenching in fluorescence intensity (Figure 1).^31^ In order to study the effect of added hexane, we recorded the absorption spectra of 5 μM solutions of **PDI-1** in 100% CHCl_3_ and gradually increased the hexane content. As shown in Figure 1c (inset), the absorbance at 553 nm shows a decreasing trend with an increase in hexane content and a gradual blue-shift from 553 to 533 nm. Both these observations indicated the formation of *H*-*type* aggregates of **PDI-1** in hexane solution.^33^

**Figure 2:**
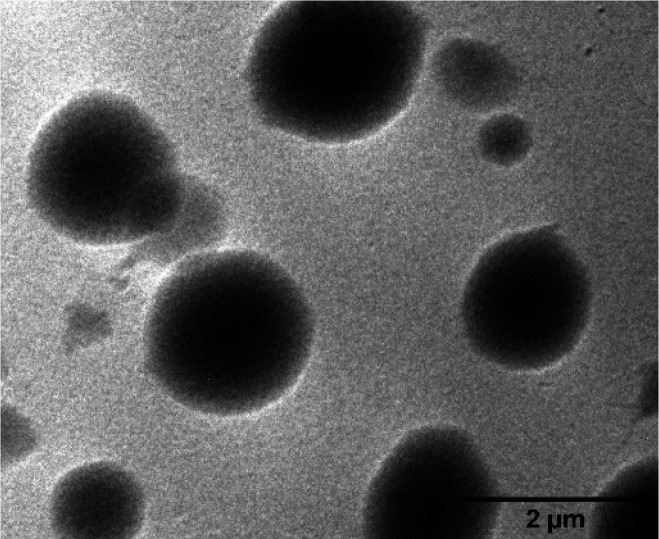
TEM image of **PDI-1** aggregated vesicles in hexane (10 μM).

Further information regarding the type of aggregation was obtained from electron microscopy studies. It was found that vesicles were formed by these *H*-aggregates with an average diameter of 962 ± 644 nm (Figure 2 and Figure S5). A *d*-spacing value of 3.11 Å in the PXRD analysis indicated the distorted π–π stacking nature of perylene cores of **PDI-1** in hexane solution, leading to the formation of these vesicles (Figure S6).^34,35^ The distortion in π–π stacking was caused by the long alkyl chain substituents present at the core positions of **PDI-1**.^36^ In addition to the distorted π–π interaction, IR analysis proved that the hydrogen bonding type interaction was absent during the aggregation process as vibrational bands of N–H and amide-linked C=O did not shift significantly in hexane compared to the disaggregated form of **PDI-1** in CHCl_3_ solution (Figure S7).

With an increase in the concentration of **PDI-1** in the hexane solution from 5 to 100 μM, significant changes in both absorption and fluorescence spectra were observed. A distinguishable shoulder peak emerged in the longer wavelength region of the absorption spectrum and fluorescence intensity decreased gradually as shown with arrows in Figure S8. This concentration-dependent self-assembly study of **PDI-1** in hexane, showed that the self-aggregation process of **PDI-1** is intermolecular, and the extent of the *H*-*type* aggregation increased with increasing the concentration.^37^

### Gelation Studies

Further, the ability of gel formation by **PDI-1** in hexane was examined by increasing the concentration of **PDI-1**. To our dismay, the compound did not form a gel even when the concentration was increased to 10 mM. The reason that this may be due to the distortion in π–π stacking of perylene moieties of **PDI-1**. It restricted the molecules to arrange along one direction to form long fiber-like structures.^35,36^ However, **PDI-1** formed gels in MCH and toluene. These gels are formed with critical gelation concentrations (CGCs) of 3.3 mM each. The formation of these organogels was confirmed by the “vial inversion test” (Figure S9).^38,39^ The gelation ability of **PDI-1** in different solvents is summarized in Table 2.

**Table 2:** Gelation Test of **PDI-1** in Different Solvents

solvent	gel/precipitate	CGC (mM)
hexane	precipitate	-
MCH	gel	3.3
toluene	gel	3.3

These gels were characterized in their dried form (xerogels) using electron microscopy and PXRD. To perform these analyses, the organogels were dried under high-vacuum to evaporate the solvents.

### Morphologies and Structures

The three-dimensional (3-D) network-like morphology of these xerogels was observed under a scanning electron microscope (SEM).^40−43^ The xerogel of MCH appeared as a 3-D cross-linked network-like cage structure, and toluene xerogel came out as regularly arranged 3-D clusters (Figure S10).

**Figure 3:**
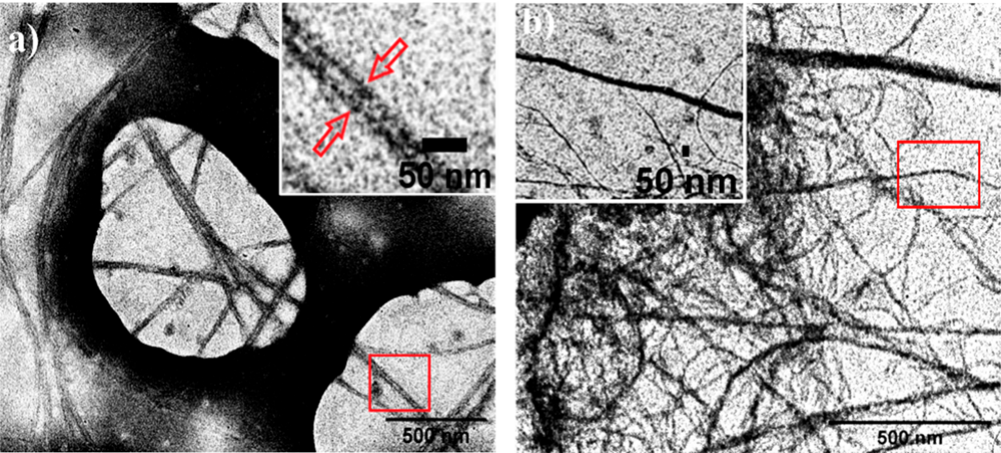
TEM images of (a) nanotubes from toluene xerogel (inset: enlarged image of a single nanotube, showing its side walls with arrows) and (b) nanofibers from MCH xerogel (inset: enlarged image of a single nanofiber).

However, xerogels revealed contrasting structures when examined under the transmission electron microscope (TEM). The hierarchical self-aggregates of toluene xerogel appeared as nanotubes (Figure 3a). These elongated nanotubes have an average diameter of 23 ± 6 nm (Figure S11) and several hundred nanometers in length. To the best of our knowledge, this is one of the very few reports of an organogel composed of nanotubes. On the contrary, the MCH xerogel consisted of nanofibers of an average diameter of 12 ± 3 nm extending to several micrometers in length (Figure 3b and Figure S12). The 3-D network-like morphology and trapping of solvents in these dense networks of nanostructures demonstrate the feature of the organogels.

### Molecular Packing

**Figure 4:**
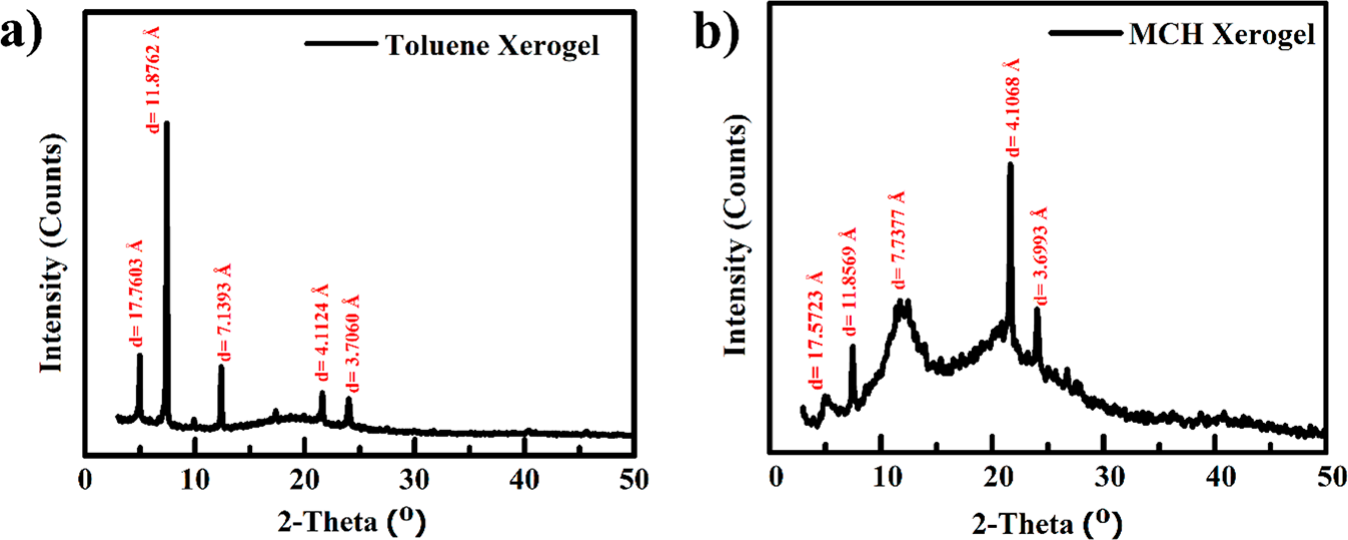
XRD patterns of (a) toluene and (b) MCH xerogel.

The molecular packing of these xerogels is revealed by PXRD analysis with their difference in the nanostructures. The PXRD analysis of toluene xerogel showed a peak at 24° (*d* = 3.7 Å), indicating the interlayer spacing between two perylene cores due to the π–π stacking of the **PDI-1** in the small molecular form (Figure 4a).^35,36^ The peak at 21.6° (*d* = 4.1 Å) belongs to the liquid-like packing order of long alkyl chains located at the core positions of **PDI-1**.^44,45^ The third-order diffraction peak of 21.6° appears at 7.4° (*d* = 11.87 Å) belonging to the (010) plane. The *d*-value ratio 1:√3 of peaks at 21.6° (*d* = 4.1 Å) and 12.4° (*d* = 7.13 Å) indicates hexagonal packing of **PDI-1** molecules in toluene organogel.^29,32,33^ The fifth order peak of π–π at 4.9° (*d* = 17.76 Å) in the small angle region belongs to the (001) plane of the nanotube.^44,48^ The higher-order peaks in PXRD analysis proved that the gelation is caused due to hierarchical self-aggregation. In MCH xerogel, a peak at 24° (*d* = 3.7 Å) belongs to π–π stacking between two perylene cores of **PDI-1** (Figure 4b).^35,36^ The first, second, and third-order diffraction peaks appeared at 21.6° (*d* = 4.1 Å), 11.4° (*d* = 7.7 Å), and 7.4° (*d* = 11.8 Å), respectively, revealing a liquid-like packing order of long alkyl chains.^44,46,47,49^ These results corroborated the lamellar packing of **PDI-1** in MCH organogel.^50^

### Hydrogen Bonding

In addition to the strong π–π interaction, the hydrogen bonding-based interactions are also envisaged between the carbonyl oxygen atom and the amide hydrogen atom to drive the aggregation process in the **PDI-1** system.^45,51^ Considering this, the IR spectroscopic analyses of **PDI-1** organogels was performed compared with its disaggregated form in CHCl_3_ solution. Compared with the N–H vibration band of disaggregated **PDI-1** in CHCl_3_ at 3291 cm^–1^ (Figure S7b), the N–H vibration band of toluene organogel disappeared in the range of 3100–3500 cm^–1^ (Figure S7c).^44,49,50^ The overlapping of vibration bands belongs to the amide carbonyl group with the carbonyl group vibrations of imide positions at 1660 cm^–1^ in CHCl_3_, are shifted to 1604 cm^–1^ (Figure S7c). These significant observations are the result of hydrogen-bonding-directed π–π interaction of the **PDI-1** in the toluene organogel.^44,52^ For the MCH organogel, the N–H vibration band and C=O vibration peaks belonging to the amide linkage emerged at 3160 and 1634 cm^–1^, respectively, in the low-frequency region, indicating hydrogen bonding directed self-aggregation (Figure S7d).^44,49,52^ The maximum shift of the amide carbonyl group stretching frequency to the lower-frequency region in toluene organogel as compared with the MCH organogel has proved the stronger hydrogen bonding of **PDI-1** in former than latter.

### Rheological Study

**Figure 5:**
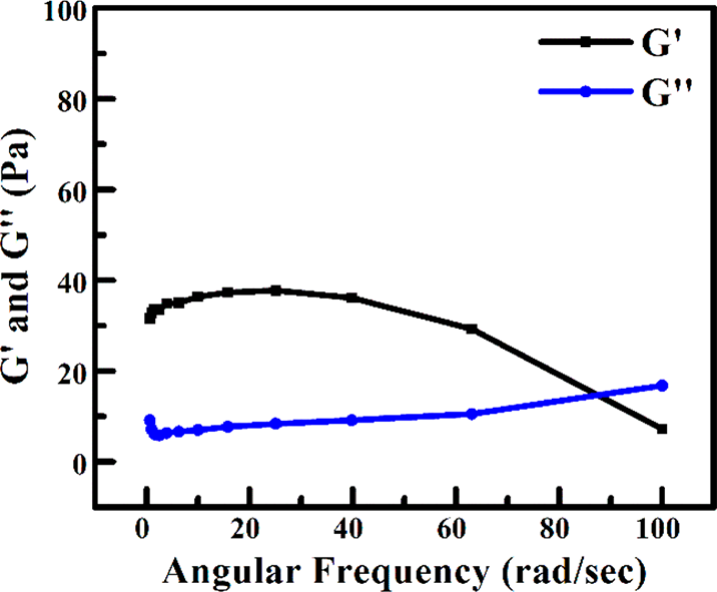
Rheology study of toluene organogel with variation of frequency.

The rheological properties of gels largely influence the potential applications in many technical areas like biotechnological, medical, and products such as foods, fuels, and ceramics. Concerning the mechanical behavior of an organogel comprising 3-D cross-linked nanotubes, the rheological analysis was carried out with the stepwise increase of oscillation frequency from 0 to 100 rad/s while keeping a constant strain value of 0.05%. The storage modulus (*G*′) and loss modulus (*G*′′) represent the elastic and viscous behavior of gel, respectively. Organogels are expected to have a *G*′ invariant with frequency and that it would be higher than *G*′′. It has been seen that both these conditions are met for the toluene organogel with a sol/gel transition point at 83 rad/s (Figure 5). The magnitude of *G*′ is also <10 times that of *G*′′. The sol/gel transition point is the phase transition point where the gel state changed its character to liquid state. These results are indicating it as a weak organogel.^49,53,54^ Additionally, the organogel of MCH was not strong enough to undergo rheological analysis. It turned into a precipitate while performing the analysis.

### Conclusions

**Figure 6:**
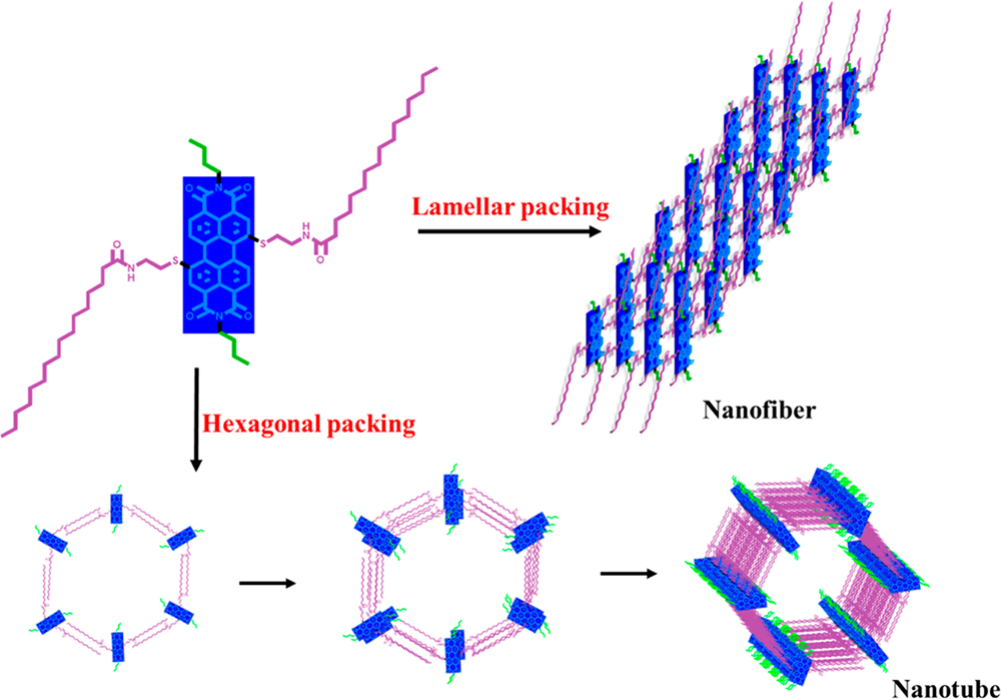
Schematic representation of molecular packing of nanostructures.

In conclusion, we formed organogels consisting of nanotubes and nanofibers from a newly synthesized core substituted PDI derivative, and this is to the best of our knowledge, the first time that perylene diimide based organogel composed of nanotubes was obtained. The hydrogen bonding directed π–π stacking of **PDI-1** led to hierarchical self-assembly and formed gels in respective solvents as evidenced by UV/vis, fluorescence, and IR spectroscopic studies. The hierarchical self-assembly mechanism was proved by PXRD analysis showing higher-order molecular packing of these organogels. The lamellar packing of **PDI-1** molecules guided to the formation of nanofibers, whereas hexagonal columnar packing provoked these molecules to self-assemble into nanotubes (Figure 6). The extent of hydrogen bonding and the difference in molecular packing of these organogels made a difference in their nanostructures. On the contrary, lack of hydrogen bonding directed and hierarchical self-assembly, prevented **PDI-1** molecules to form a gel in hexane though maximal changes were observed in the absorption and fluorescence spectra. The hollow cylindrical morphology of nanotubes addresses the appropriate necessity for molecular orientation than nanofibers and vesicles. In these π-stacked arrays formed from a π-electronic organic system like PDI can admit directional transports of energy and charge carriers. We are considering these features of this dominant material for fabricating optoelectronic devices in the near future.

## Methods

Analytical grade solvents were purchased from Avra Synthesis Pvt. Ltd. Other Chemicals and reagents were used from Merck, Sigma-Aldrich, and Alfa Aesar. The compounds were purified using a 63–210 μm silica gel in column chromatography. Samples were confirmed by ^1^H and ^13^C NMR of 400 MHz-Bruker in CDCl_3_ solution. Bruker UltrafleXtreme MALDI-TOF mass spectrometer and Q-Exactive TM-BenchTop-LC-HRMS were used to obtain the mass value of the new compound. On LAMBDA 365 UV/vis spectrophotometer absorption spectra were recorded in solution form. Fluorescence spectrometer-HITACHI F-7000 was helped in measuring the fluorescence spectra of samples with an excitation wavelength of 560 nm. The molecular packing of self-assembled aggregates and xerogels were analyzed by Powder-X-ray Diffractometer-(Bruker, D8, advance) on a glass substrate and powder form, respectively. The hydrogen bonding type interaction was characterized by FT-IR spectroscopy (Thermo Fisher Scientific Nicolet iS10). The aggregates and organogels were spin-coated (1000 rpm) on a glass substrate, and the solvents were removed using a high-vacuum to study their morphology by FE-SEM (Thermo Fischer FEI QUANTA 250 FEG with a voltage range of 5–30 kV). The exact structure of the aggregates and xerogels was identified by a TEM-FEI-TecnaiG220 Twin using a carbon-coated copper grid of size 300 mesh. An Anton Paar302 rheometer equipped with a steel-coated parallel-plate geometry (25 mm of diameter) was used for the rheological analysis of an organogel at 0.05% strain.

### Preparation of **cys-PDI-cys**

DiBrPDI (50 mg, 0.076 mmol), cetyltrimethylammonium bromide (166 mg, 0.456 mmol), and potassium carbonate (63 mg, 0.456 mmol) were put together in a round-bottom flask and high vacuumed to make it air free. THF (10 mL) was added to the above mixture and stirred at room temperature for 30 min with continuous purging of nitrogen gas until the mixture was dissolved. After that, the cysteamine hydrochloride (52 mg, 0.456 mmol) was added to the mixture, and an immediate color change was observed from orange to dark-red purple color. The reaction mixture was kept for stirring at room temperature for 24 h under inert atmosphere. The completion of reaction was monitored by TLC plate. Then THF was evaporated in a Rota evaporator, and the mixture was washed with water and chloroform. The organic layer (chloroform) was collected and dried in a Rota evaporator to get the crude solids, which were later purified by column chromatography using 3–5% methanol/chloroform solvents to get the desired product **cys-PDI-cys** as purple solid (37 mg, 74%).

^1^H NMR (400 MHz, CDCl_3_): 8.72 (s, 2H, perylene-H), 8.69 (d, *J* = 5.5 Hz, 2H, perylene-H), 8.57 (d, *J* = 8.0 Hz, 2H, perylene-H), 4.14 (t, *J* = 7.4 Hz, 4H, N(CH_2_(CH_2_)_2_CH_3_)), 3.20 (t, *J* = 6.2 Hz, 4H, CH_2_–S), 2.87 (t, *J* = 6.2 Hz, 4H, NH_2_-(CH_2_)_2_S), 1.66 (m, 8H, N(CH_2_(CH_2_)_2_CH_3_) and CH_2_–NH_2_), 1.40 (m, 4H, N(CH_2_(CH_2_)_2_CH_3_)), 0.94 (t, *J* = 7.3 Hz, 6H, N(CH_2_(CH_2_)_2_CH_3_)).

^13^C NMR (100 MHz, CDCl_3_): 163.47, 137.53, 134.35, 133.23, 132.60, 131.57, 129.21, 128.47, 125.62, 122.28, 121.76, 40.76, 40.62, 40.24, 30.35, 20.54, 14.00.

MS (MALDI-TOF): *m*/*z* calculated for C_36_H_36_N_4_O_4_S_2_ [M^+^]: 652.22; found [M + H] ^+^: 653.31.

HR-MS (ESI-TOF, positive mode): *m*/*z* calculated for C_36_H_36_N_4_O_4_S_2_ [M^+^]: 652.2178; found [M + H] ^+^: 653.2255.

### Preparation of **PDI-1**

Palmitic acid (26 mg, 0.101 mmol) was dissolved in chloroform (10 mL) with the addition of thionyl chloride (32 mg, 0.276 mmol) and a catalytic amount of dimethylformamide. The mixture was stirred at room temperature for 3 h. After that, the chloroform was evaporated in a Rotary evaporator, and the reaction mixture was again dissolved in chloroform (10 mL) followed by addition of **cys-PDI-cys** (20 mg, 0.031 mmol) and trimethyl amine (14 mg, 0.143 mmol). The mixture was stirred at room temperature for 24 h, and the completion of the reaction was monitored by a TLC. The reaction mixture was washed with water, and organic phase (chloroform) was evaporated in rota evaporator to get the crude product. Then, it was purified by column chromatography using 0.5–1% methanol/chloroform solvents affording the required product **PDI-1** as a purple solid (14 mg, 69%).

^1^H NMR (400 MHz, CDCl_3_): 8.76 (d, *J* = 8 Hz, 2H, perylene-H), 8.71 (s, 2H, perylene-H), 8.59 (d, *J* = 8.1 Hz, 2H, perylene-H), 5.76 (t, *J* = 8.3 Hz, 2H, HN-CO), 4.15 (t, 4H, *J* = 8.0 Hz, N(CH_2_(CH_2_)_2_CH_3_)), 3.25 (m, 4H, S(CH_2_)_2_NHCO), 2.27 (t, *J* = 7.5 Hz, 4H, CH_2_–S), 1.87 (t, *J* = 7.5 Hz, 4H, NHCOCH_2_), 1.68 (m, 8H, NHCO(CH_2_)_14_CH_3_), 1.56 (m, 8H, NHCO(CH_2_)_14_CH_3_), 1.42 (m, 8H, N(CH_2_(CH_2_)_2_CH_3_), 1.18 (m, 36H, NHCO(CH_2_)_14_CH_3_)), 0.94 (t, *J* = 7.3 Hz, 6H, N(CH_2_(CH_2_)_2_CH_3_)), 0.80 (t, *J* = 6.7 Hz, 6H, NHCO(CH_2_)_14_CH_3_).

^13^C NMR (100 MHz, CDCl_3_): 173.78, 159.86, 134.88, 133.90, 133.10, 132.29, 131.23, 129.41, 128.63, 127.25, 122.11, 121.90, 32.07, 29.84, 29.80, 29.74, 29.59, 29.50, 29.41, 29.39, 29.23, 28.37, 25.63, 24.89, 22.83, 20.54, 14.26, 13.99.

MS (MALDI-TOF): *m*/*z* calculated for C_68_H_96_N_4_O_6_S_2_ [M^+^]: 1129.64; found [M] ^+^: 1129.79.

HR-MS (ESI-TOF, positive mode): *m*/*z* calculated for C_68_H_96_N_4_O_6_S_2_ [M^+^]: 1129.6430, found [M] ^+^: 1129.6329.

### Gelation Test

In a glass vial, both solvent and compound are heated until the mixture gets dissolved. Then the dissolved solution is allowed to cool down to room temperature for 30 min, and the gel is confirmed by the “vial inversion test”.

## Supporting Information Available

NMR data (ZIP)
Supporting figures (PDF)
