# Supplementary material for: Organogels Fabricated from Self-Assembled Nanotubes Containing Core Substituted Perylene Diimide Derivative
Source: ACS Omega. 2022 Jun 14;7(25):21932–8. doi: 10.1021/acsomega.2c02210 (PMC9245106; doi:10.1021/acsomega.2c02210)
Supplement: Supplementary file 1 — ao2c02210_si_001.zip [file ao2c02210_si_001.zip › FID for publication/cys-PDI-cys/13C/pdata/1/email_VITCHENNAI40219_71_1.pdf]

Signature SIF VIT VELLORE
GS-PM-121

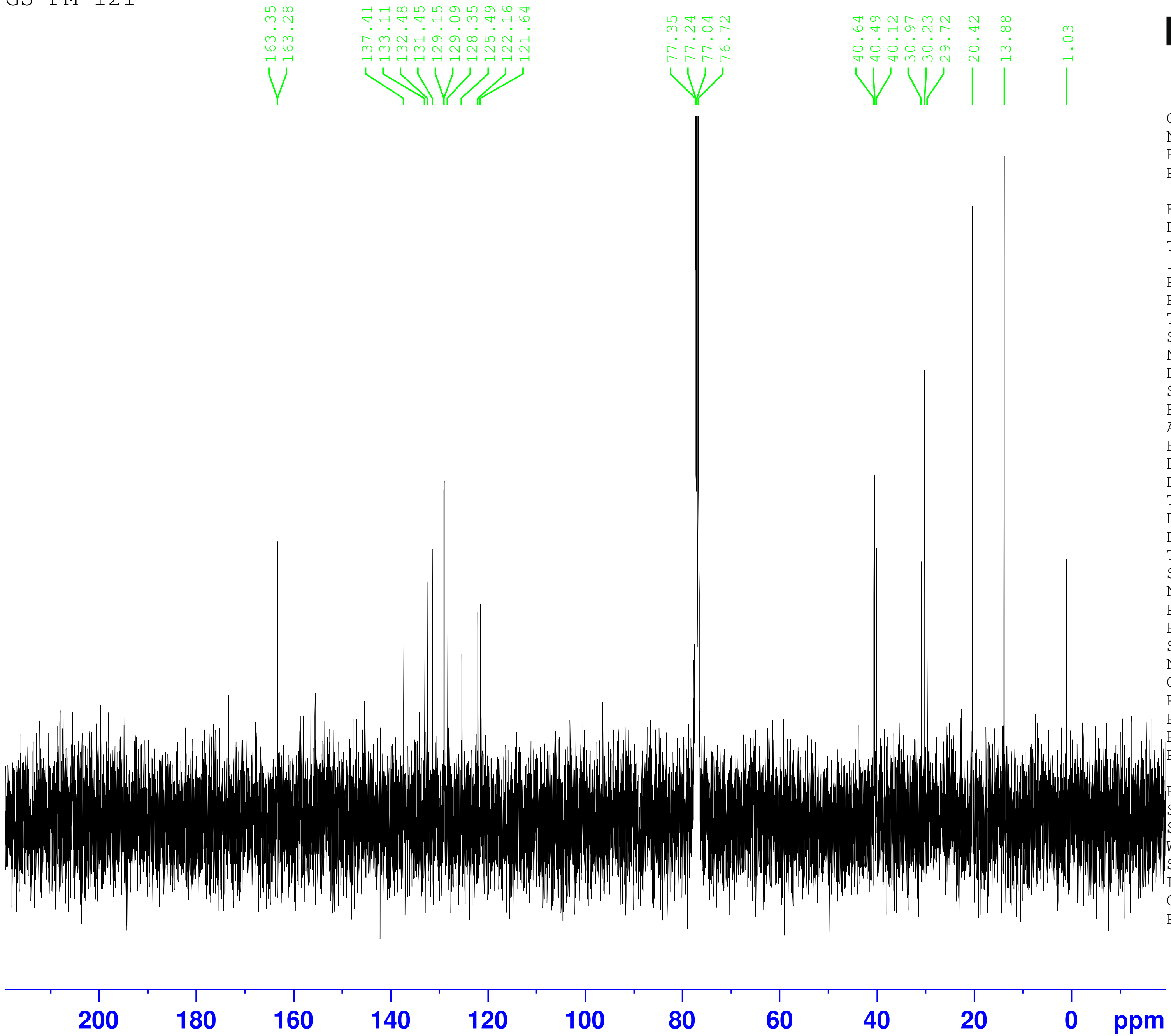

BRUKER

Current Data Parameters

NAME	VITCHENNAI40219	
EXPNO	71	
PROCNO	1	

F2 - Acquisition Parameters

Date_	20190211	
Time	10.01	h
INSTRUM	spect	
PROBHD	Z108618_0505 (	
PULPROG	zgpg30	
TD	65536	
SOLVENT	CDCl3	
NS	512	
DS	4	
SWH	24038.461	Hz
FIDRES	0.733596	Hz
AQ	1.3631488	sec
RG	127.79	
DW	20.800	usec
DE	6.50	usec
TE	304.4	K
D1	2.00000000	sec
D11	0.03000000	sec
TD0	1	
SFO1	100.6550186	MHz
NUC1	13C	
P1	9.80	usec
PLW1	58.00000000	W
SFO2	400.2596010	MHz
NUC2	1H	
CPDPRG[2	waltz16	
PCPD2	90.00	usec
PLW2	16.00000000	W
PLW12	0.38716000	W
PLW13	0.19474000	W

F2 - Processing parameters

SI	32768	
SF	100.6449542	MHz
WDW	EM	
SSB	0	
LB	1.00	Hz
GB	0	
PC	1.40	
