# Supplementary material for: Organogels Fabricated from Self-Assembled Nanotubes Containing Core Substituted Perylene Diimide Derivative
Source: ACS Omega. 2022 Jun 14;7(25):21932–8. doi: 10.1021/acsomega.2c02210 (PMC9245106; doi:10.1021/acsomega.2c02210)
Supplement: Supplementary file 1 — ao2c02210_si_001.zip [file ao2c02210_si_001.zip › FID for publication/cys-PDI-cys/1H/pdata/1/email_VITCHENNAI40219_70_1.pdf]

Signature SIF VIT VELLORE
GS-PM-121

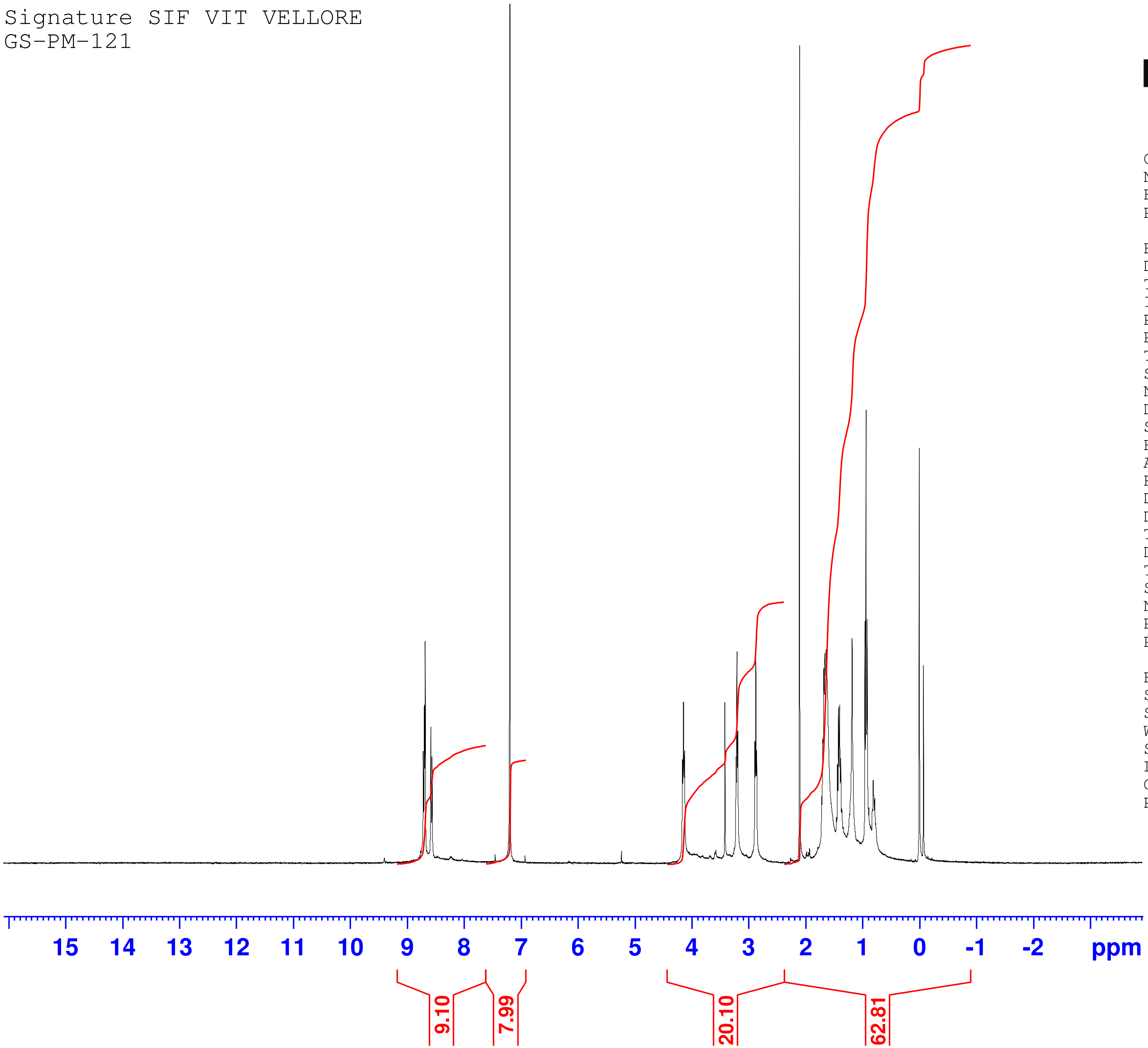

BRUKER

Current Data Parameters
NAME VITCHENNAI40219
EXPNO 70
PROCNO 1

F2 - Acquisition Parameters
Date_ 20190211
Time 9.31 h
INSTRUM spect
PROBHD Z108618_0505 (
PULPROG zg30
TD 65536
SOLVENT CDCl3
NS 16
DS 2
SWH 8012.820 Hz
FIDRES 0.244532 Hz
AQ 4.0894465 sec
RG 143.73
DW 62.400 usec
DE 6.50 usec
TE 303.6 K
D1 1.00000000 sec
TD0 1
SFO1 400.2604716 MHz
NUC1 1H
P1 14.00 usec
PLW1 16.00000000 W

F2 - Processing parameters
SI 65536
SF 400.2580356 MHz
WDW EM
SSB 0
LB 0.30 Hz
GB 0
PC 1.00
