# Supplementary material for: Organogels Fabricated from Self-Assembled Nanotubes Containing Core Substituted Perylene Diimide Derivative
Source: ACS Omega. 2022 Jun 14;7(25):21932–8. doi: 10.1021/acsomega.2c02210 (PMC9245106; doi:10.1021/acsomega.2c02210)
Supplement: Supplementary file 1 — ao2c02210_si_001.zip [file ao2c02210_si_001.zip › FID for publication/PDI-1/13C/pdata/1/email_VITC40121_32_1.pdf]

Signature SIF VIT VELLORE
PM-126

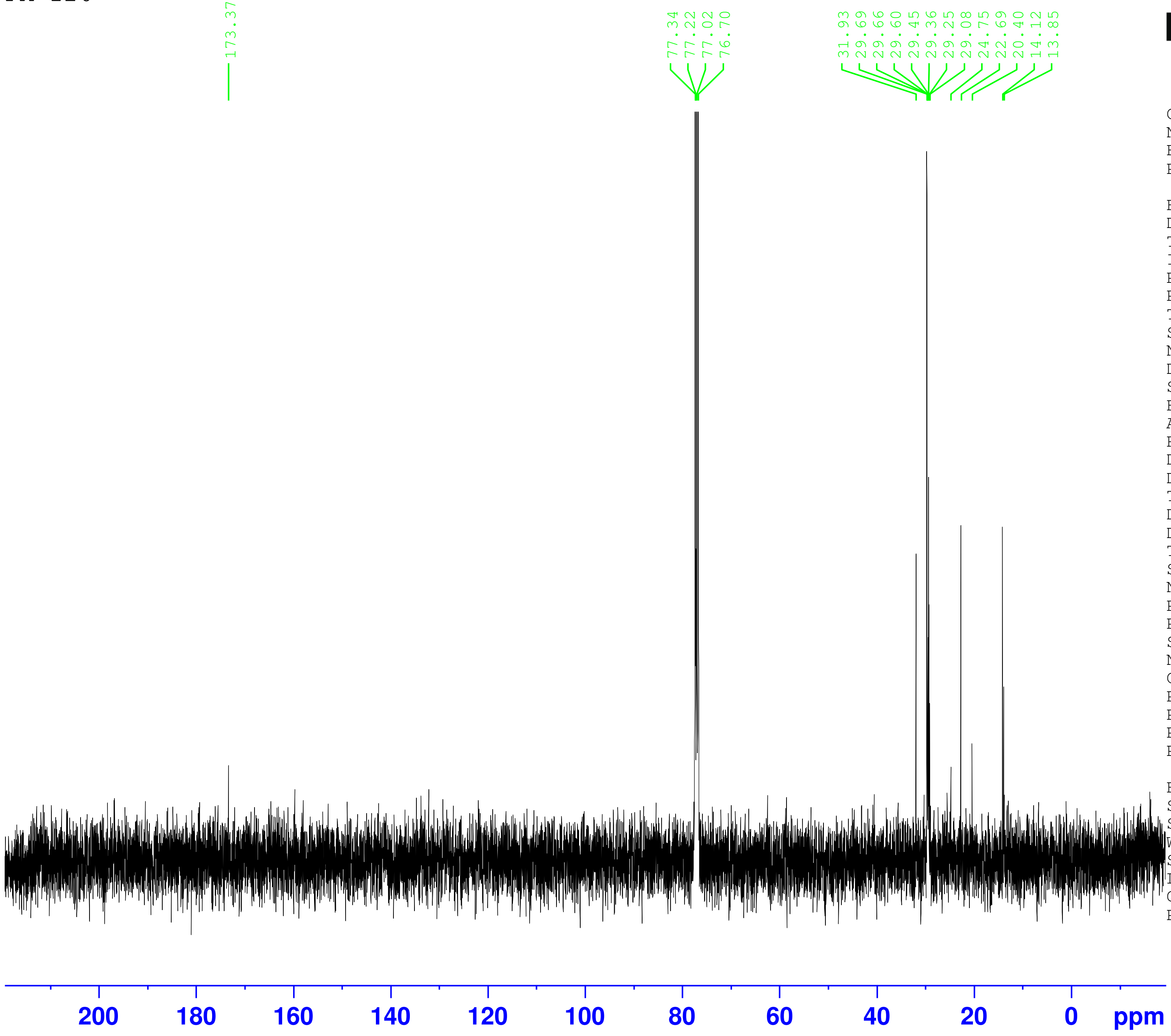

BRUKER

Current Data Parameters
NAME VITC40121
EXPNO 32
PROCNO 1

F2 - Acquisition Parameters
Date_ 20210124
Time 12.56 h
INSTRUM spect
PROBHD Z108618_0505 (
PULPROG zgpg30
TD 65536
SOLVENT CDCl3
NS 512
DS 4
SWH 24038.461 Hz
FIDRES 0.733596 Hz
AQ 1.3631488 sec
RG 156.91
DW 20.800 usec
DE 6.50 usec
TE 332.0 K
D1 2.00000000 sec
D11 0.03000000 sec
TD0 1
SFO1 100.6550185 MHz
NUC1 13C
P1 10.52 usec
PLW1 58.00000000 W
SFO2 400.2596010 MHz
NUC2 1H
CPDPRG[2 waltz16
PCPD2 90.00 usec
PLW2 16.00000000 W
PLW12 0.39104000 W
PLW13 0.19668999 W

F2 - Processing parameters
SI 32768
SF 100.6449540 MHz
WDW EM
SSB 0
LB 1.00 Hz
GB 0
PC 1.40
