# Supplementary material for: Organogels Fabricated from Self-Assembled Nanotubes Containing Core Substituted Perylene Diimide Derivative
Source: ACS Omega. 2022 Jun 14;7(25):21932–8. doi: 10.1021/acsomega.2c02210 (PMC9245106; doi:10.1021/acsomega.2c02210)
Supplement: Supplementary file 1 — ao2c02210_si_001.zip [file ao2c02210_si_001.zip › FID for publication/PDI-1/1H/pdata/1/email_VITC40121_31_1.pdf]

Signature SIF VIT VELLORE
PM-126

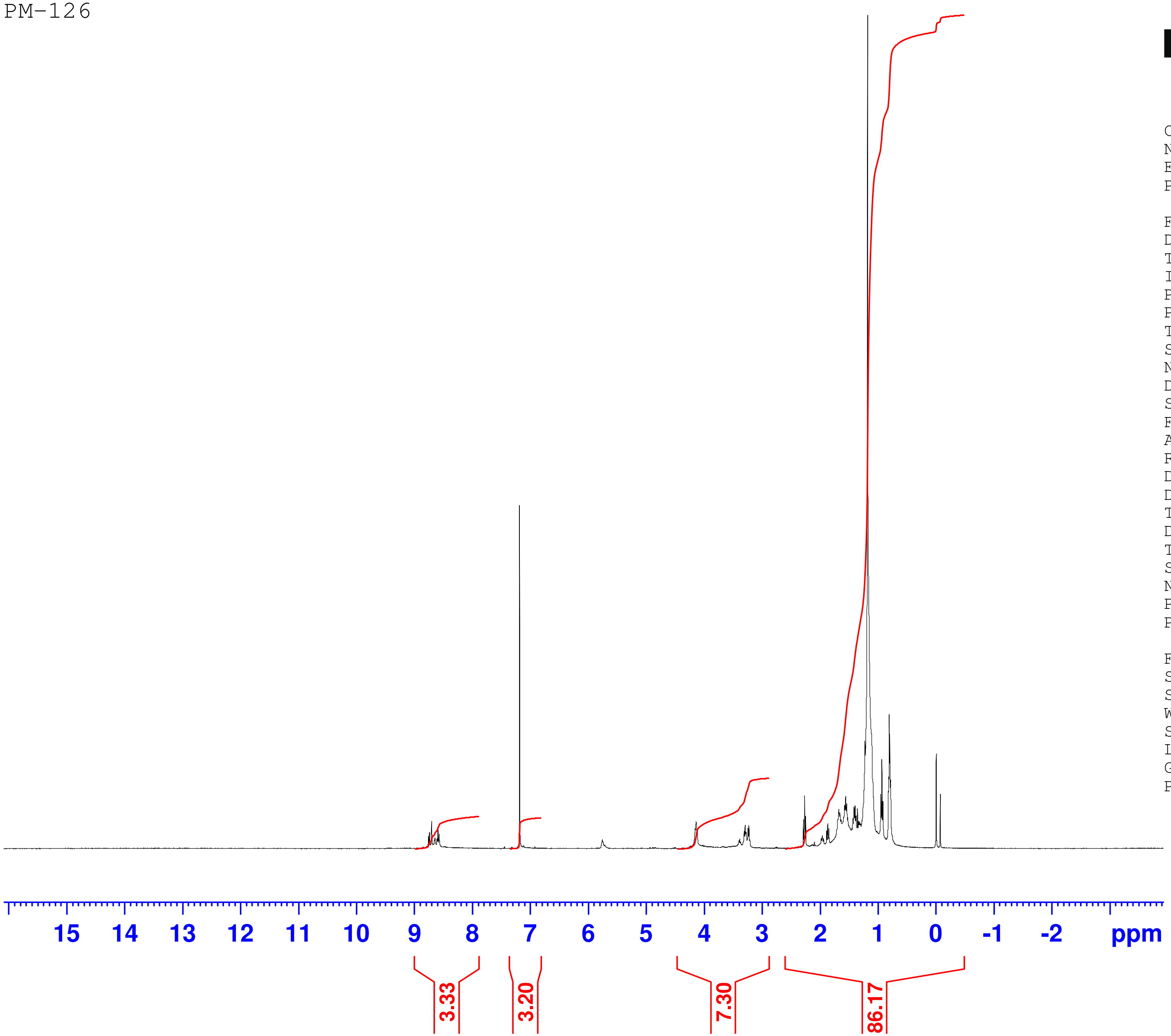


BRUKER

Current Data Parameters

| | |
|---|---|
| NAME | VITC40121 |
| EXPNO | 31 |
| PROCNO | 1 |

F2 - Acquisition Parameters

| | |
|---|---|
| Date_ | 20210124 |
| Time | 12.25 h |
| INSTRUM | spect |
| PROBHD | Z108618_0505 ( |
| PULPROG | zg30 |
| TD | 65536 |
| SOLVENT | CDCl3 |
| NS | 32 |
| DS | 2 |
| SWH | 8012.820 Hz |
| FIDRES | 0.244532 Hz |
| AQ | 4.0894465 sec |
| RG | 143.73 |
| DW | 62.400 usec |
| DE | 6.50 usec |
| TE | 331.5 K |
| D1 | 1.00000000 sec |
| TD0 | 1 |
| SFO1 | 400.2604716 MHz |
| NUC1 | 1H |
| P1 | 14.07 usec |
| PLW1 | 16.00000000 W |

F2 - Processing parameters

| | |
|---|---|
| SI | 65536 |
| SF | 400.2580367 MHz |
| WDW | EM |
| SSB | 0 |
| LB | 0.30 Hz |
| GB | 0 |
| PC | 1.00 |
