# Supplementary material for: Organogels Fabricated from Self-Assembled Nanotubes Containing Core Substituted Perylene Diimide Derivative
Source: ACS Omega. 2022 Jun 14;7(25):21932–8. doi: 10.1021/acsomega.2c02210 (PMC9245106; doi:10.1021/acsomega.2c02210)
Supplement: Supplementary file 2 — ao2c02210_si_002.pdf [file ao2c02210_si_002.pdf]

**Supporting Information for:**

# Organogels fabricated from self-assembled nanotubes containing Core substituted Perylene diimide derivative.

PRAJNA MOHARANA and G. SANTOSH *
Division of Chemistry, School of Advanced Sciences, Vellore Institute of Technology, Chennai– 600127, INDIA

**Contents**

1. $^{1}H$ NMR data
2. UV/Vis spectra in different organic solvents
3. UV/Vis vs Fluorescence spectra
4. Scanning electron microscope (SEM) image and histogram
5. Powder X-ray diffraction (PXRD) analysis
6. IR analysis
7. Concentration dependent study
8. Organogels
9. Scanning electron microscope (SEM) images of xerogels
10. Size distribution of nanostructure using histogram

### *Characterization*

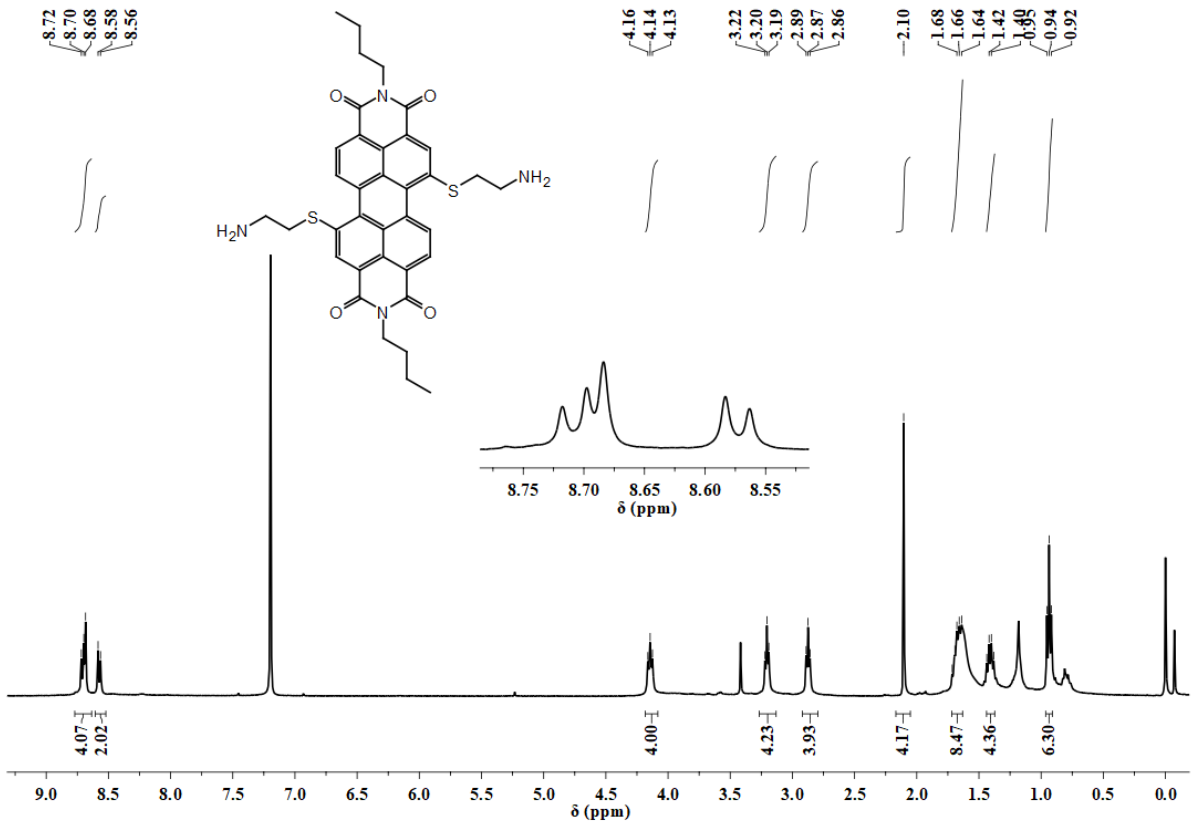


**Figure S1.** $^{1}$H NMR (400 MHz, $CDCl_3$) spectrum of **cys-PDI-cys**.

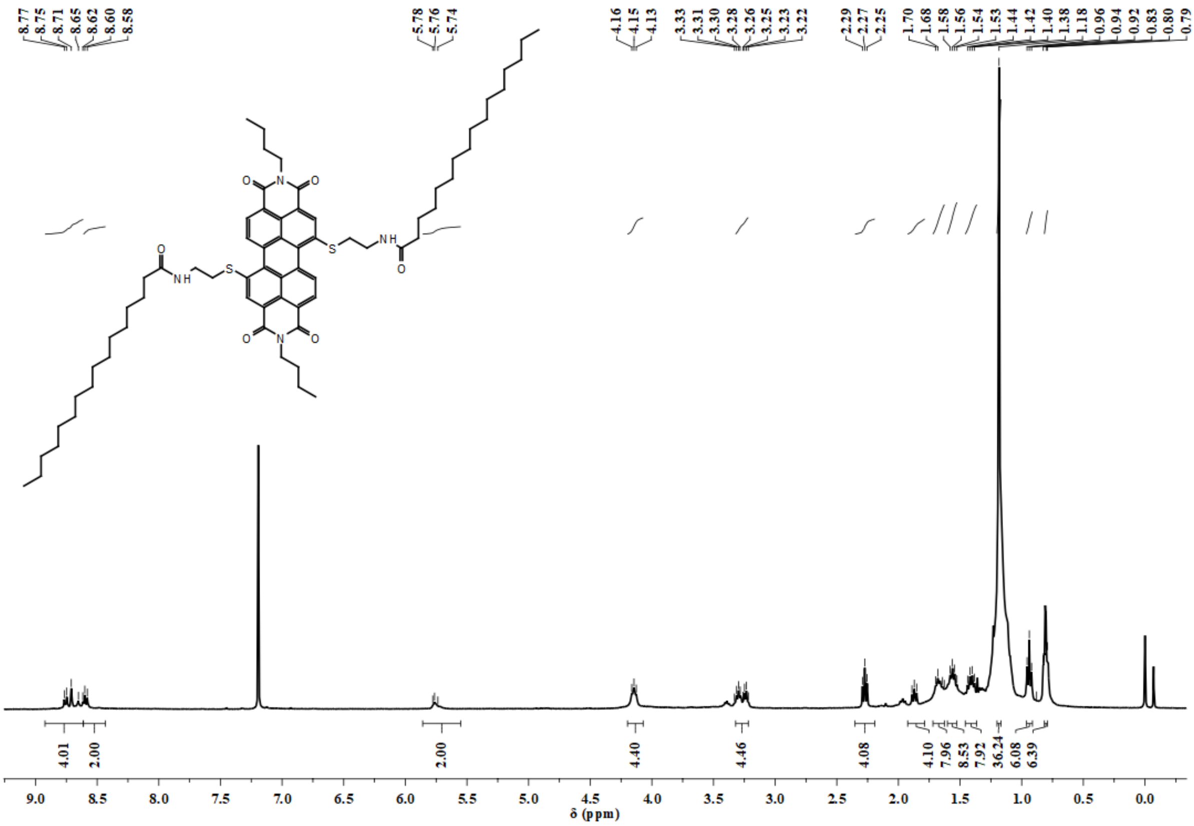

**Figure S2.** $^{1}$H NMR (400 MHz, $CDCl_3$) spectrum of **PDI-1**.

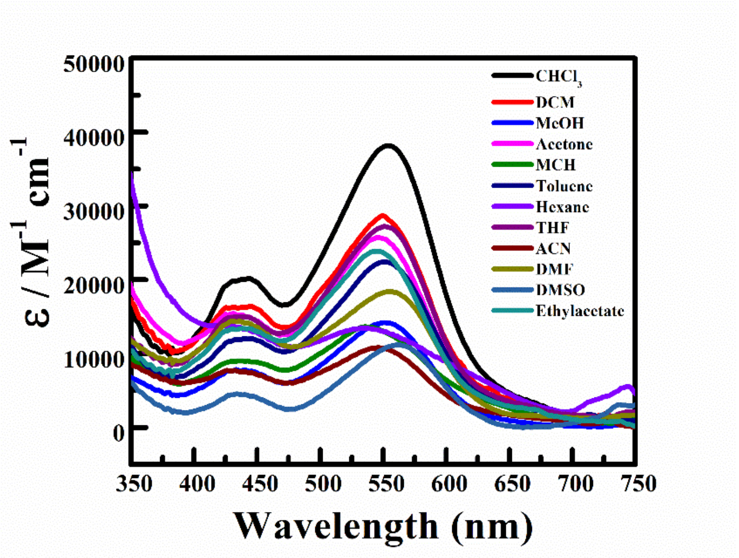


**Figure S3**. Absorption spectra of **PDI-1** in different organic solvents ($5\mu M$).

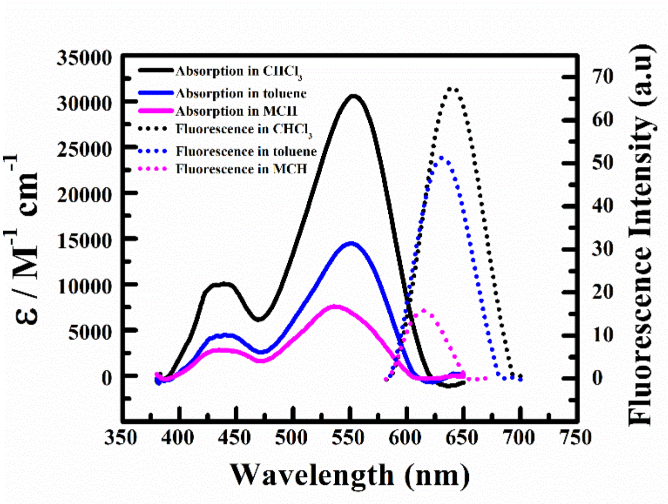


**Figure S4.** Absorption and fluorescence spectra of **PDI-1** in $CHCl_3$, toluene and MCH ($5\mu M$).

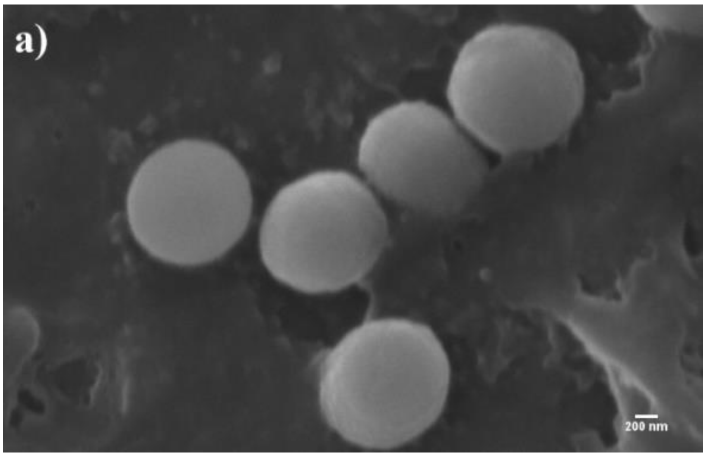


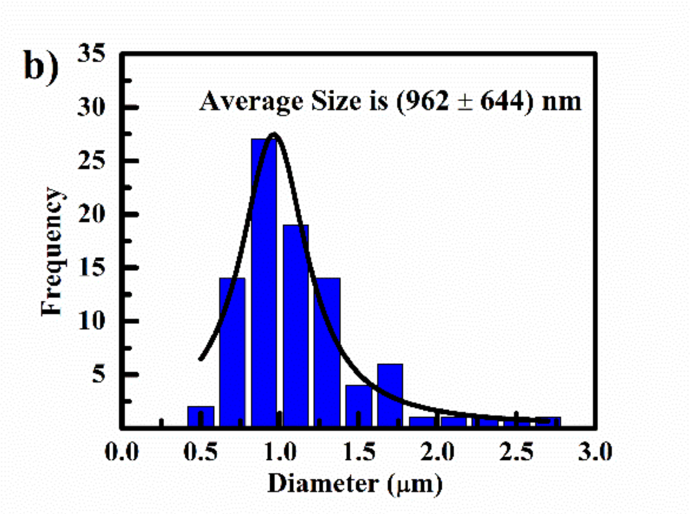

**Figure S5.** a) SEM image of **PDI-1** spherical aggregates in hexane (10$\mu M$), and b) Size distribution histogram of vesicle diameter.

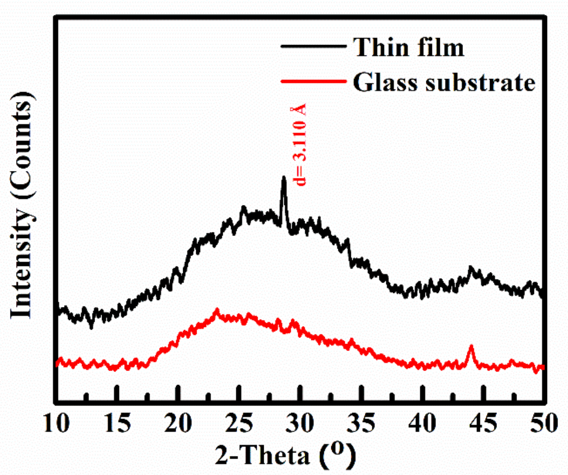


**Figure S6.** XRD patterns of spherical aggregates of **PDI-1** in hexane solution (10$\mu M$).

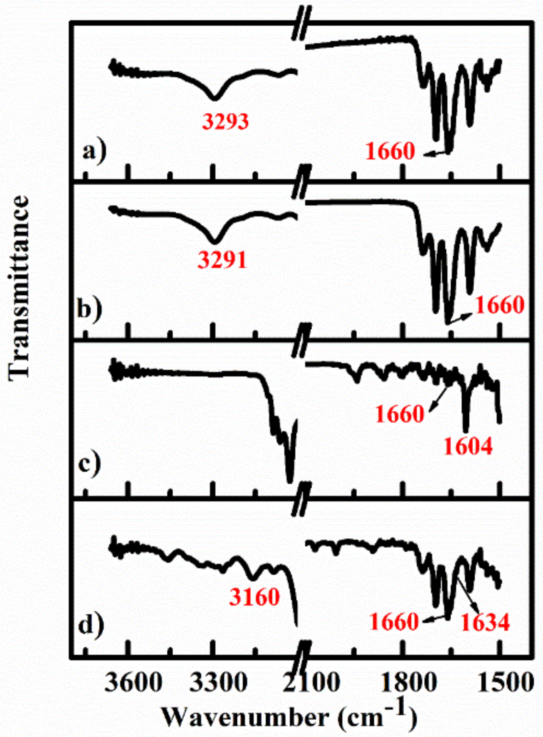


**Figure S7.** IR spectra of **PDI-1** in a) hexane and b) $CHCl_3$. IR spectra of organogels of c) toluene, and d) MCH.

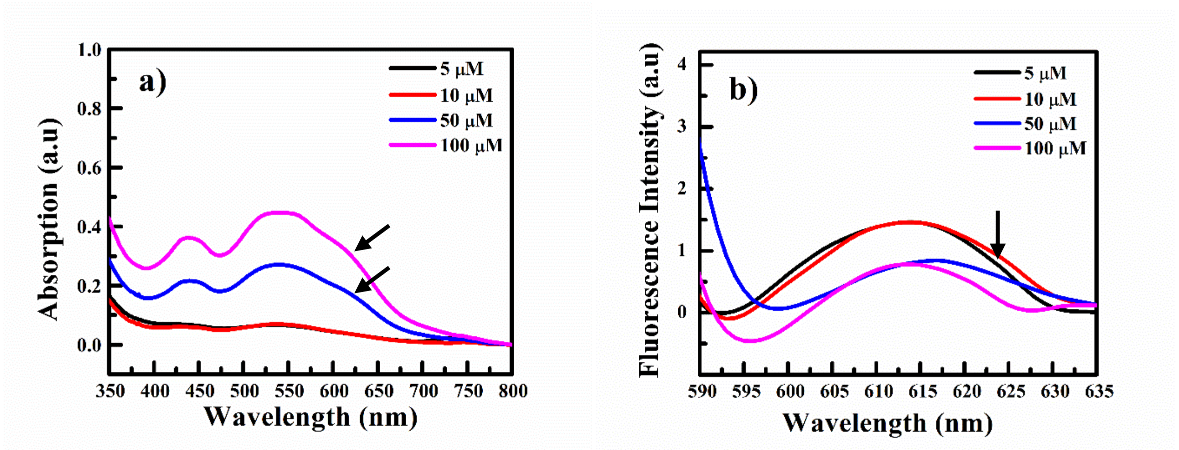


**Figure S8.** a) Absorption spectra and b) Fluorescence spectra of **PDI-1** in hexane in different concentrations ($5\mu M$, $10\mu M$, $50\mu M$, and $100\mu M$).

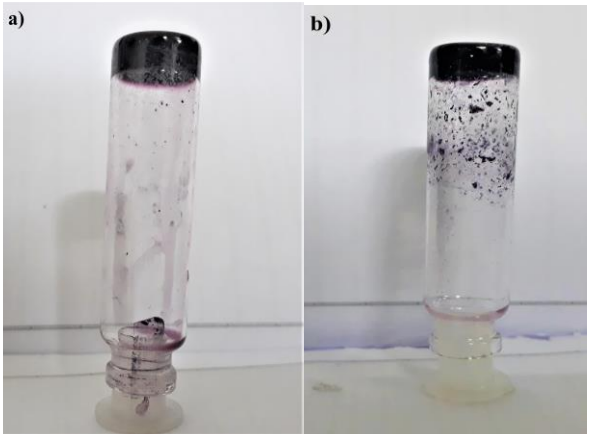


**Figure S9. PDI-1** organogels of a) toluene and b) MCH.

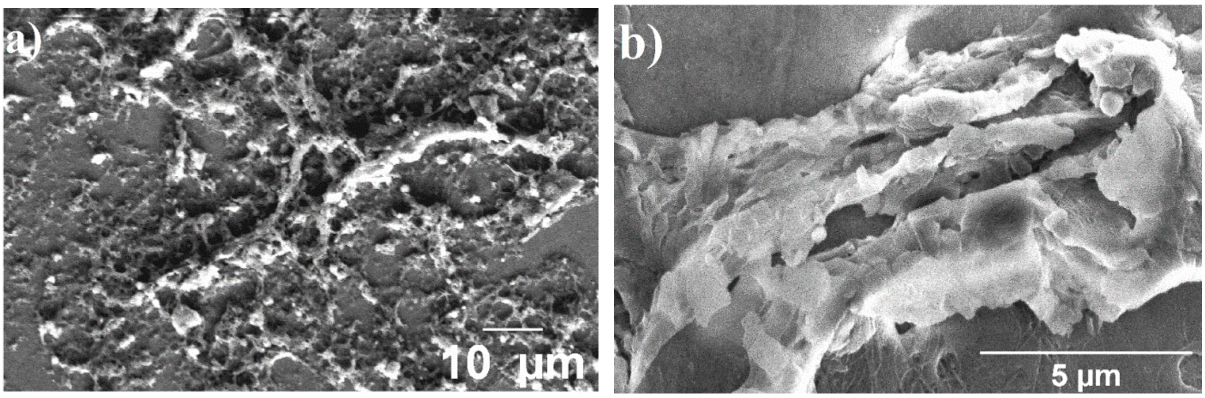

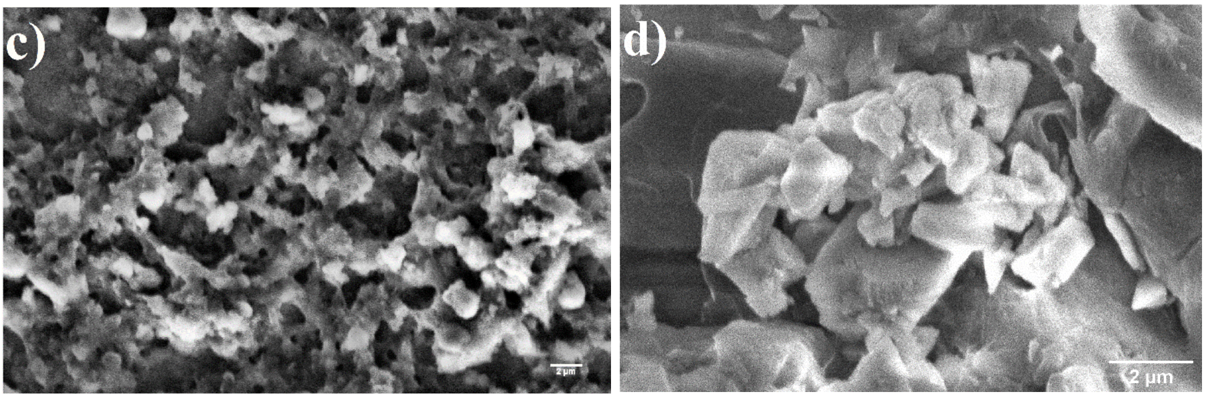


**Figure S10.** SEM images of xerogel of a) MCH, scale bar 10$\mu m$, b) toluene, scale bar 5$\mu m$, c) MCH, scale bar 2$\mu m$ and d) toluene, scale bar 2$\mu m$

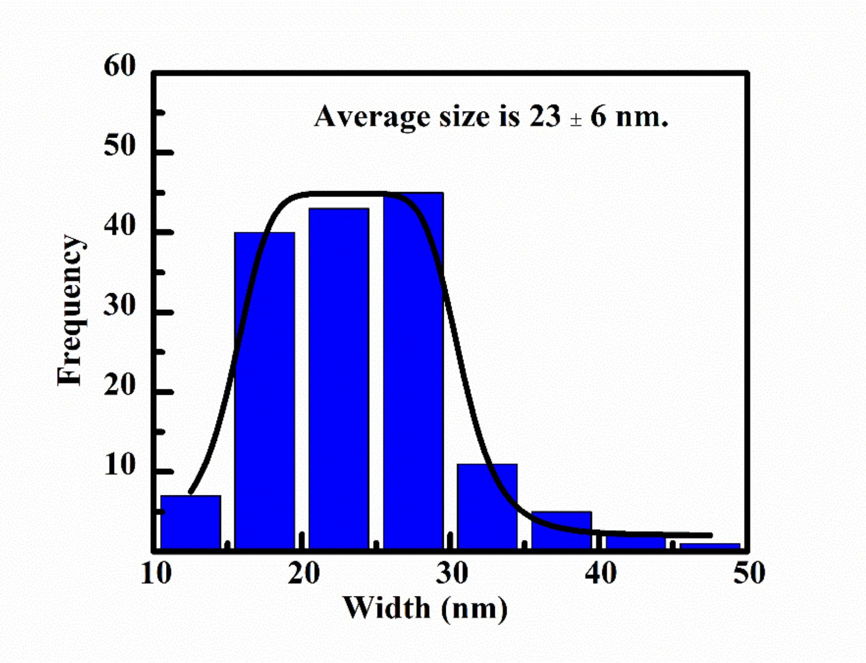


**Figure S11.** Size distribution histogram of nanotubes from toluene xerogel.

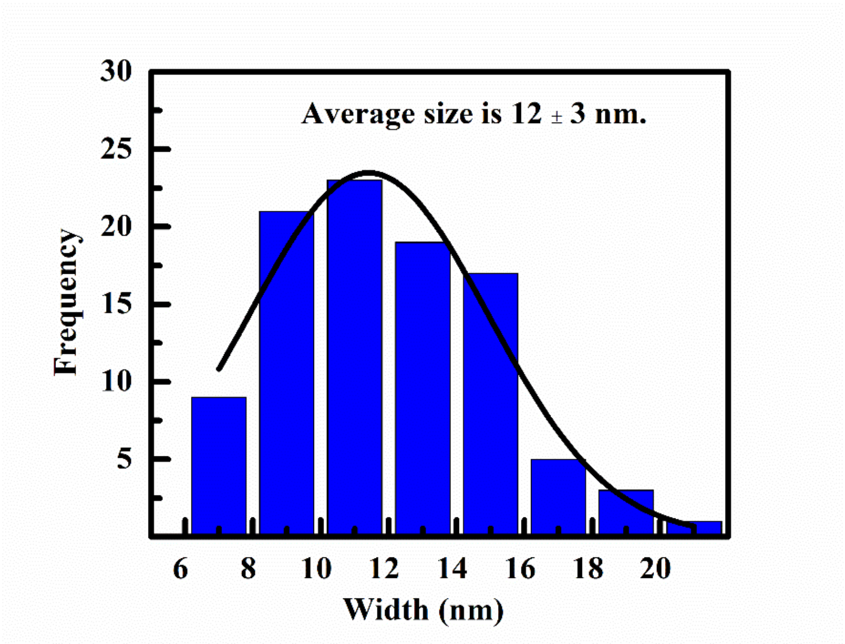


**Figure S12.** Size distribution histogram of nanofibers from MCH xerogel.
